# Effects of Pilates Programs on Body Composition, Flexibility, Muscular Strength, Balance, Functional Performance and Respiratory Function in Adult Women: A Systematic Review and Meta-Analysis

**DOI:** 10.3390/sports14090407

**Published:** 2026-09-15

**Authors:** Čedomir Bajčetić, Vojin Rudović, Milica Novaković, Mirela Vještica, Roksanda Atanasov, Vuk Ralić, Marko Manojlović, Patrik Drid

**Affiliations:** Faculty of Sport and Physical Education, University of Novi Sad, 21000 Novi Sad, Serbia

**Keywords:** exercise intervention, body composition, flexibility, muscular strength, balance, functional performance, respiratory function

## Abstract

Although Pilates has become an increasingly popular form of exercise among adult women, previous systematic reviews have often combined heterogeneous populations and outcomes, leaving the specific effects of structured Pilates programs on health-related physical fitness in this population insufficiently clarified. This systematic review and meta-analysis examined the effects of structured Pilates programs on health-related physical fitness components in adult women, following PRISMA 2020 guidelines with a prospectively registered PROSPERO protocol. A literature search was conducted in PubMed, Web of Science, and Google Scholar up to 21 January 2026. Of 751 records identified, 12 studies (2007–2025, approximately 540 female participants) were included in the final synthesis, and meta-analyses were performed using Review Manager 5.4.1 with a random-effects model. Pilates interventions produced statistically significant improvements in body mass index (MD = −1.22; 95% CI: −1.83 to −0.61; *p* < 0.001), body weight (MD = −3.99 kg; 95% CI: −6.30 to −1.67; *p* = 0.0007), and sit-and-reach flexibility (SMD = 0.69; 95% CI: 0.43 to 0.94; *p* < 0.001), whereas no significant pooled effects emerged for body fat percentage, waist circumference, waist-to-hip ratio, lean body mass, handgrip strength, or vertical jump. Narrative synthesis suggested additional positive effects on muscular strength, endurance, balance, functional abilities, and respiratory parameters. Pilates appears to be a safe, effective method for improving selected body composition parameters and flexibility in adult women, though findings warrant cautious interpretation given heterogeneity in study design, intervention protocols, and methodological quality. This study was supported by the Provincial Secretariat for Higher Education and Scientific Research (grant 142-451-3098).

## 1. Introduction

Modern societies are increasingly characterized by sedentary lifestyles, largely driven by technological development and reduced requirements for daily physical movement. Although physical activity may no longer be essential for immediate survival in the same way as in earlier periods of human development, it remains a fundamental biological and health-related need. Regular movement contributes to the optimal functioning of the human organism and is closely associated with physical, psychological, and social well-being. In contrast, insufficient physical activity is related to a higher risk of numerous adverse health outcomes, including metabolic disorders, reduced functional capacity, musculoskeletal problems, and impaired mental health [1]. In this context, structured exercise programs that are safe, accessible, and adaptable to different populations have become increasingly important in health promotion and preventive exercise practice.

Pilates is a mind–body exercise method developed by Joseph Pilates in the early twentieth century. Initially, it was primarily practiced by dancers and athletes, but it has gradually become widely used among the general population [2]. Pilates is based on principles such as control, precision, centering, concentration, breathing, and movement flow. Through controlled and coordinated exercises, Pilates aims to improve trunk stability, muscular strength and endurance, flexibility, postural control, and movement efficiency [3,4]. Different Pilates modalities, including mat Pilates, reformer Pilates, and combined approaches, are commonly applied in exercise programs. Due to its relatively low-impact nature and adaptability, Pilates may be particularly suitable for adult women with different levels of physical activity and fitness.

Health-related physical fitness components are especially relevant in adult women because they are closely associated with chronic disease prevention, functional independence, and quality of life. Parameters such as body composition, muscular strength, flexibility, balance, functional performance, and respiratory function represent important indicators of health status and physical functioning. Unfavorable changes in these components, including increased body fat, reduced muscle strength, impaired balance, and decreased mobility, may contribute to a greater risk of metabolic disorders, falls, disability, and reduced well-being, particularly with advancing age [5]. Therefore, exercise interventions aimed at improving these components are of practical importance for maintaining health, preventing functional decline, and supporting active lifestyles in women.

Previous research has suggested that Pilates may have beneficial effects on several components of physical fitness; however, the available findings remain heterogeneous. Cruz-Ferreira et al. [6] reported strong evidence for improvements in flexibility and dynamic balance, as well as moderate evidence for improvements in muscular endurance, but emphasized the generally low methodological quality of the included studies. Aladro-Gonzalvo et al. [7] concluded that there was limited quantitative evidence supporting a positive effect of Pilates on body composition and highlighted methodological limitations such as inadequate study designs, poor standardization of measurement procedures, and insufficient control of nutritional status. Furthermore, Fernández-Rodríguez et al. [8] showed that Pilates training may improve flexibility, strength, and functional capacity in older adults, while also reducing the risk of falls. Individual intervention studies provide further evidence across a broad range of health-related fitness outcomes. Pilates training has been shown to improve trunk strength, muscular endurance, and flexibility in sedentary adult women [9], and lumbar strength and flexibility in postmenopausal women [10]. Beneficial effects on body composition have also been reported in sedentary, middle-aged, and obese women [11,12,13], while individual studies have documented improvements in respiratory function and joint mobility [14], functional performance [15], and physical and psychological well-being [16] in healthy women across different age groups. Although some studies suggest positive effects of Pilates training programs on physical fitness and body composition, the available evidence remains inconsistent, and the effects in healthy individuals without chronic diseases have been insufficiently explored [7,17,18]. Nevertheless, previous reviews often included mixed populations, different age groups, various health conditions, and diverse intervention protocols, which makes it difficult to determine the specific effects of Pilates in healthy adult women. Therefore, the present study aimed to systematically review and quantitatively synthesize the available evidence on the effects of structured Pilates programs on health-related physical fitness components in adult women. The practical significance of this study lies in providing clearer evidence for exercise prescription and health promotion practice. Based on the available theoretical and empirical evidence, it was hypothesized that structured Pilates programs would have positive effects on selected health-related physical fitness components, particularly flexibility, muscular fitness, balance, functional performance, and body composition parameters, in adult women.

## 2. Materials and Methods

### 2.1. Study Design

This study was conducted as a systematic review with meta-analysis in accordance with the PRISMA 2020 guidelines. The PRISMA 2020 checklist was completed to ensure transparent and comprehensive reporting of all review stages. The research protocol was prospectively registered in the International Prospective Register of Systematic Reviews database PROSPERO (CRD420261290568). The predefined methodology included the search strategy, eligibility criteria, study selection process, data extraction procedure, risk of bias assessment, and data synthesis plan.

### 2.2. Search Strategy

A literature search was conducted in PubMed, Web of Science (WoS), and Google Scholar to identify relevant studies published up to 21 January 2026, with no restriction regarding year of publication. Only studies published in English were considered eligible. Database-specific search strategies were developed using combinations of keywords, synonyms, controlled vocabulary, and Boolean operators (AND and OR), adapted to the search interface and indexing system of each database.

In PubMed, the search strategy combined MeSH terms and title/abstract terms related to Pilates, women, and physical fitness and health-related outcomes. The following search strategy was applied:

(“Pilates” [Mesh] OR Pilates [Title/Abstract] OR “Pilates method” [Title/Abstract] OR “Pilates training” [Title/Abstract]) AND (“Women” [Mesh] OR women [Title/Abstract] OR female* [Title/Abstract]) AND (“Body Composition” [Mesh] OR “Muscle Strength” [Mesh] OR “body composition” [Title/Abstract] OR flexibility [Title/Abstract] OR “muscle strength” [Title/Abstract] OR balance [Title/Abstract] OR “functional performance” [Title/Abstract] OR “respiratory function” [Title/Abstract]) AND English [Language] AND (“1 January 1900” [Date—Publication]: “21 January 2026” [Date—Publication]).

In Web of Science, the search was conducted using topic-related keywords covering Pilates, women, and the predefined physical fitness and health-related outcomes:

(Pilates OR “Pilates method” OR “Pilates training”) AND (women OR female) AND (“body composition” OR “muscle strength” OR flexibility OR balance OR “functional performance” OR “respiratory function”).

In Google Scholar, a simplified keyword-based search strategy was used because of the database’s different search functionality and indexing structure:

Pilates AND women AND (“body composition” OR flexibility OR “muscle strength” OR balance OR “functional performance” OR “respiratory function”).

In addition, backward citation tracking was performed by manually screening the reference lists of included studies and relevant systematic reviews, while forward citation tracking was used to identify newer articles citing the included studies. All identified records were imported into EndNote, where duplicates were removed before the screening process. The literature search and study identification process were conducted in accordance with the pre-registered PROSPERO protocol, with a minor expansion of the data sources to identify additional relevant literature.

### 2.3. Selection Process

**Figure 1:**
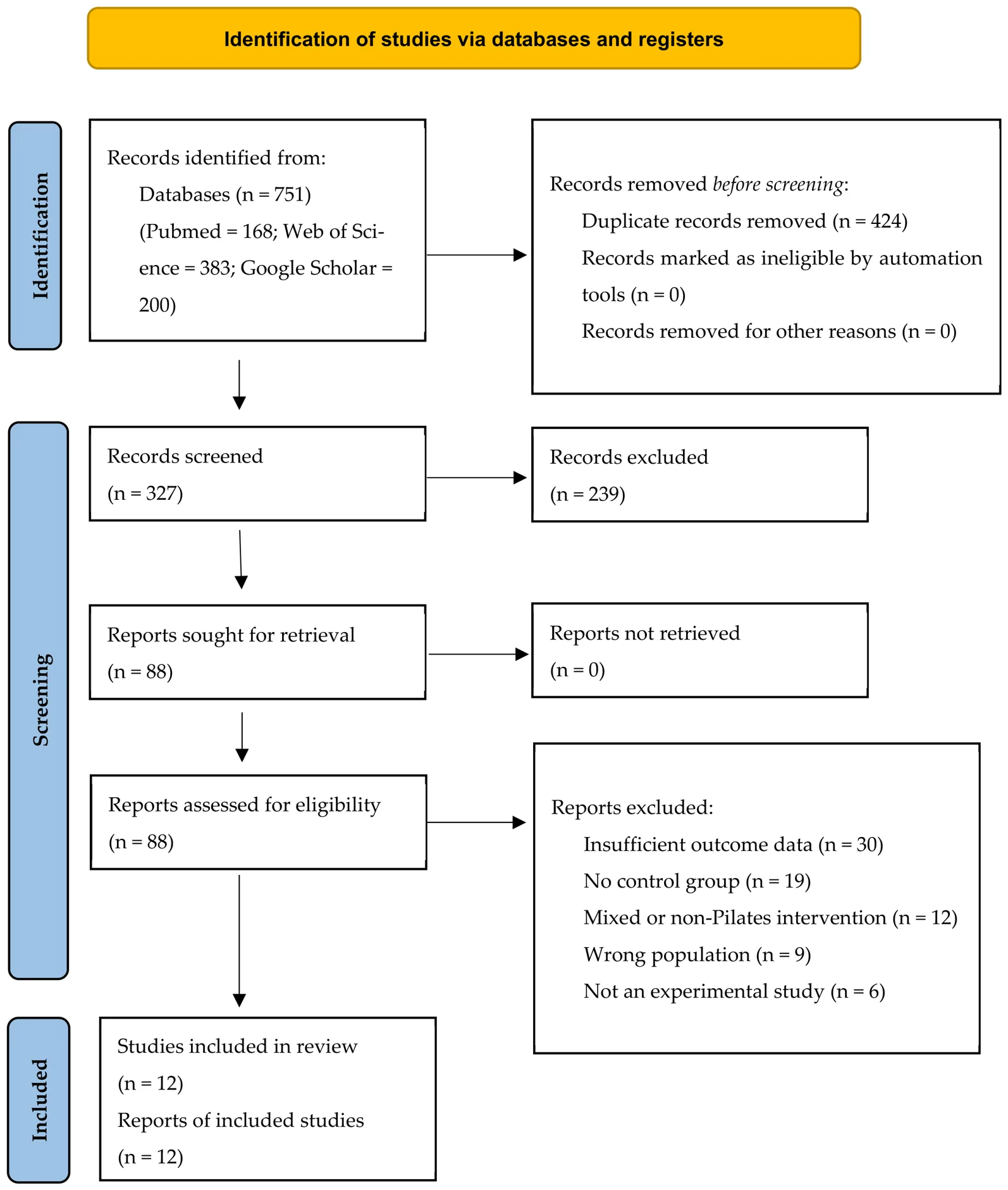
PRISMA diagram.

Two reviewers (ČB and VR) independently conducted the study selection process. The selection included title and abstract screening, assessment of records sought for retrieval, and full-text evaluation of potentially eligible studies. Disagreements between reviewers were resolved through discussion, and if consensus could not be reached, a third reviewer (MM) was consulted to make the final decision (see Figure 1).

### 2.4. Eligibility Criteria

The inclusion and exclusion criteria were defined according to the PICOS framework: Population, Intervention, Comparison, Outcomes, and Study design. The review included studies involving adult women, regardless of their level of physical activity, who did not have acute or chronic diseases that could substantially influence the response to exercise intervention. Eligible interventions included structured Pilates programs, including mat Pilates, reformer Pilates, or combined approaches, regardless of intervention duration or weekly training frequency. Studies were required to include a control or comparison group, such as an inactive control group, usual daily activity, or alternative forms of low- to moderate-intensity physical activity. Eligible studies had to report at least one quantitatively measurable health-related physical fitness outcome, including body composition, muscle strength and endurance, flexibility, balance, functional abilities, respiratory function, or cardiovascular parameters. Randomized controlled trials and quasi-experimental studies with a control or comparison group were included, whereas studies without a control group, observational studies, review articles, and case studies were excluded.

### 2.5. Data Extraction

Data extraction was performed independently by two reviewers (CB and VR) using a predefined and standardized data extraction table. Extracted data included author(s), year of publication, study design, participant characteristics, sample size, group allocation, intervention characteristics, control condition, measurement instruments, and reported outcomes. The extracted data were organized into tables to improve clarity and transparency. Specifically, the general characteristics of the included studies are presented in Table 1, the characteristics of the Pilates interventions are presented in Table 2, and the outcomes not eligible for quantitative synthesis are presented in Table 3. General characteristics of the included studies were presented separately from intervention characteristics and outcomes not eligible for quantitative synthesis. Outcomes were categorized according to their suitability for meta-analysis; outcomes reported in at least two studies and measured comparably were included in quantitative synthesis, while outcomes that could not be pooled due to methodological heterogeneity or insufficient reporting were summarized narratively.

### 2.6. Risk of Bias

Risk of bias was systematically assessed for all included studies according to study design. Randomized controlled trials were assessed using the Risk of Bias 2 tool (RoB 2) across five domains: bias arising from the randomization process, bias due to deviations from intended interventions, bias due to missing outcome data, bias in measurement of the outcome, and bias in selection of the reported result. The results of the risk-of-bias assessment for randomized controlled trials are presented in Table 4. Non-randomized studies were assessed using the Risk Of Bias In Non-randomized Studies of Interventions tool (ROBINS-I) across seven domains: bias due to confounding, bias in selection of participants, bias in classification of interventions, bias due to deviations from intended interventions, bias due to missing data, bias in measurement of outcomes, and bias in selection of the reported result. The results of the risk-of-bias assessment for non-randomized studies are presented in Table 5. The overall risk-of-bias judgement was determined based on the highest level of risk identified across the relevant domains. The assessment was performed independently by three reviewers (CB, VR, and MN), and disagreements were resolved through discussion. When consensus could not be reached, an additional reviewer (MM) was consulted.

### 2.7. Data Analysis

Meta-analysis was performed using Review Manager software version 5.4.1. Outcomes reported in at least two studies were included in quantitative synthesis, while outcomes reported in fewer than two studies or measured using highly heterogeneous instruments were summarized narratively. For each outcome, post-intervention data were extracted and used for analysis. For outcomes measured using the same units, mean difference (MD) with 95% confidence intervals (CI) was calculated. For outcomes measured using different instruments or units, the standardized mean difference (SMD) with 95% CI was used. SMD values were interpreted as trivial (<0.2), small (0.2 to <0.5), moderate (0.5 to <0.8), and large (≥0.8) according to Cohen [19]. Given the expected clinical and methodological heterogeneity related to participant characteristics, study design, and intervention protocols, a random-effects model was applied. Statistical heterogeneity was assessed using the I^2^ statistic and interpreted as low when I^2^ was ≤50% and high when I^2^ was >50% in accordance with Higgins et al. Statistical significance was set at *p* < 0.05, and all meta-analysis results were presented using forest plot diagrams generated in Review Manager 5.4.1.

### 2.8. Certainty of Evidence

The overall certainty of evidence was assessed using the Grading of Recommendations Assessment, Development and Evaluation (GRADE) approach. The certainty of evidence was classified as high, moderate, low, or very low. The initial certainty of evidence was determined according to the study design, with randomized controlled trials (RCTs) initially rated as high certainty and non-randomized controlled trials (Non-RCTs) initially rated as low certainty. The certainty of evidence was assessed across five GRADE domains: (1) risk of bias, downgraded by one level when important methodological limitations were identified in the majority of included studies; (2) inconsistency, downgraded by one level when substantial statistical heterogeneity was observed (I^2^ > 50%); (3) indirectness, downgraded by one level when important differences existed between the study population, intervention, comparator, or outcome and the review question; (4) imprecision, downgraded by one level when the 95% confidence interval indicated substantial uncertainty around the pooled estimate, including wide confidence intervals or intervals crossing the line of no effect; and (5) publication bias, downgraded by one level when evidence suggestive of publication bias was identified. The certainty of evidence was upgraded when a large magnitude of effect was observed, in accordance with the GRADE approach. Two reviewers (CB and VR) independently assessed the certainty of evidence for all primary and secondary outcomes, and disagreements were resolved by consensus with a third reviewer (MM) (see Table 6).

## 3. Results

### 3.1. Study Selection

The selection was conducted in accordance with the PRISMA 2020 guidelines. During the initial search, electronic databases PubMed, Web of Science, and Google Scholar identified a total of 751 papers (PubMed = 168; Web of Science = 383; Google Scholar = 200 best of evaluated papers, which were found by a combination of keywords) (see Figure 1). After removing duplicates (*n* = 424), 327 published papers were included in the further selection process. During the title and abstract review phase, 239 papers were excluded because they did not meet the defined PICOS criteria. There remained 88 papers whose full texts were read and assessed against the inclusion criteria. After a detailed analysis of the full texts, another 76 studies were excluded due to insufficient outcome data (*n* = 30), lack of a control group (*n* = 19), wrong population (*n* = 9), mixed or non-Pilates intervention (*n* = 12), and a non-experimental study design (*n* = 6). The final number of papers included in the systematic review and meta-analysis is 12, which were entered in the data synthesis.

### 3.2. Characteristics of Included Studies

A total of 12 studies were included in this systematic review and meta-analysis, comprising randomized controlled trials (RCTs) and non-randomized controlled trials (non-RCTs). The studies were published between 2007 and 2025 and involved a total of approximately 540 female participants. The general characteristics of the included studies, including study design, participant demographics, and sample size, are presented in Table 1. As shown in Table 2, Pilates intervention protocols varied in terms of duration, weekly frequency, session length, and program characteristics. Intervention duration ranged from 5 to 12 weeks, while training frequency ranged from one to four sessions per week. Most programs were conducted two or three times per week, with sessions most commonly lasting 60 min. The interventions generally included supervised, progressive, standard, mat-based, or beginner-level Pilates programs. Control groups most frequently involved no exercise, usual lifestyle, or usual daily activity, indicating moderate heterogeneity in intervention and comparison conditions across the included studies.

Across the included studies, health-related physical components which are not included in the meta-analysis were assessed, including body composition, anthropometry, flexibility, muscle strength and endurance, balance, and functional performance. and respiratory function. A detailed overview of outcome domains, measurement instruments, and reported findings is presented in Table 3.

Overall, the included studies provide heterogeneous but complementary evidence regarding the effect of Pilates exercise programs on multiple health and performance-related outcomes in adult women.

**Table 1:** General Characteristics of Included Studies.

Author	Study Design	Population	Age (Years)	Sample (*n*)	Pilates Group/ Control Group (*n*)
Aibar-Almazán et al. (2022) [5]	RCT	Community-dwelling women	CG = 67.60 ± 9.77; PG = 70.71 ± 7.80.	109	55/54
Ceviz et al. (2025) [11]	RCT	Sedentary elderly women	CG = 58.91 ± 6.33; PG = 59.25 ± 6.15.	24	12/12
Kalkan-Balak et al. (2024) [20]	RCT	Healthy sedentary women	CG = 28.08 ± 6.00; PG = 34.16 ± 8.83; WBV = 33.25 ± 8.13.	36	12/12 + (12 WBV)
dos Reis et al. (2024) [21]	RCT	Sedentary young women	CG = 29.00 ± 7.59; PG = 27.44 ± 5.70.	32	16/16
Hushmandi et al. (2023) [13]	Non—RCT	Obese middle-aged women	CG = 37.35 ± 3.23; PG = 38.05 ± 4.37.	40	20/20
Lee et al. (2016) [10]	Non—RCT	Postmenopausal women	CG = 55.31 ± 3.16; PG = 53.00 ± 3.62.	74	45/29
Sekendiz et al. (2007) [9]	RCT	Sedentary adult females	CG = 30.8 ± 8.6; PG = 30.2 ± 6.6.	38	21/17
Vieira et al. (2017) [15]	RCT	Community-dwelling women	CG = 63.3 ± 0.91; PG = 66.0 ± 1.35.	40	21/19
Ružić (2020) [22]	RCT	Female college students	19–22 yrs., mean age was not reported.	30	15/15
Kolomiitseva et al. (2022) [14]	Non—RCT	Healthy middle-aged women	Total 45.4 ± 2.3.	32	18/14
Tolnai et al. (2016) [16]	Non—RCT	Young sedentary women	CG = 20.94 ± 1.60; PG = 22.2 ± 2.30.	50	32/18
Şavkın et al. (2017) [12]	RCT	Healthy middle-aged women	CG = 39.67 ± 6.30; PG = 43.79 ± 4.88.	37	19/18

**Table 2:** Characteristics of Pilates Intervention in Included Studies.

Author	Duration (Weeks)	Frequency (x/Week)	Session Length (min)	Program Characteristics	Control Condition
Aibar-Almazán et al. (2022) [5]	12 weeks	2 x/week	60 min	Basic–intermediate, progressive	Usual lifestyle
Ceviz et al. (2025) [11]	12 weeks	4 x/week	60–90 min	Supervised, progressive	No exercise
Kalkan-Balak et al. (2024) [20]	8 weeks	2 x/week	45 min	Supervised, progressive	No exercise
dos Reis et al. (2024) [21]	8 weeks	3 x/week	50 min	Stretching + strengthening	Stretching only
Hushmandi et al. (2023) [13]	8 weeks	3 x/week	60 min	Mat-based, progressive	No exercise
Lee et al. (2016) [10]	8 weeks	3 x/week	50–60 min	Supervised	No exercise
Sekendiz et al. (2007) [9]	5 weeks	3 x/week	60 min	Standard Pilates	No exercise
Vieira et al. (2017) [15]	12 weeks	2 x/week	60 min	Supervised	Usual activity
Ružić (2020) [22]	12 weeks	3 x/week	60 min	Progressive volume/intensity	No exercise
Kolomiitseva et al. (2022) [14]	12 weeks	3 x/week	60 min	Beginner level	No exercise
Tolnai et al. (2016) [16]	10 weeks	1 x/week	60 min	Supervised	No exercise
Şavkın et al. (2017) [12]	8 weeks	3 x/week	90 min	Standard Pilates	Usual activity

**Table 3:** Findings Not Included in Meta-Analysis.

Author	Outcome Domains and Measurement Instruments	Main Findings
Aibar-Almazán et al. (2022) [5]	Body composition: skeletal muscle mass; Functional performance/gait speed: Timed Up and Go test (TUG).	No significant between-group differences were found for skeletal muscle mass (*p* = 0.561) or TUG performance (*p* = 0.118).
Şavkın et al. (2017) [12]	Anthropometry: abdomen circumference.	Significant changes were reported in both the control group (*p* < 0.001) and Pilates group (*p* = 0.033). The direction of between-group differences should be checked in the original study.
dos Reis et al. (2024) [21]	Muscular strength: 10-RM knee extension; Muscular endurance: Sorensen test.	Significant improvements in 10 RM knee extension were observed in both the Pilates group (*p* = 0.006) and control group (*p* = 0.010). No significant improvements were found for the Sorensen test in the Pilates group (*p* = 0.768) or control group (*p* = 0.540).
Sekendiz et al. (2007) [9]	Muscular endurance/strength: curl-up test; abdomen and back strength tests.	Significant between-group differences were found for curl-up (*p* < 0.001), 60° flexion (*p* = 0.006), 60° extension (*p* = 0.001), and 120° flexion (*p* = 0.003), most likely in favour of the Pilates group. No significant difference was found for 120° extension (*p* = 0.083).
Ružić (2020) [22]	Flexibility: straddle test; Functional performance: leg lift and back leg lift.	Significant improvements were observed for the straddle test in both the Pilates group (*p* = 0.003) and control group (*p* = 0.023). No significant improvements were found for leg lift in the Pilates group (*p* = 0.052) or control group (*p* = 0.643). Back leg lift improved significantly in the Pilates group (*p* < 0.001), but not in the control group (*p* = 1.000).
Kolomiitseva et al. (2022) [14]	Respiratory function: respiratory rate, vital capacity, Stange test, Genchi test; Flexibility/ROM: prone shoulder flexion; Functional performance: side lift, roll-up with bent legs, roll-up with straight legs; Muscular strength: leg pull, push-up on single knee.	Respiratory rate did not significantly change in the Pilates group (*p* = 0.19) or control group (*p* = 0.92). Significant improvements were observed in the Pilates group for vital capacity (*p* = 0.05), Stange test (*p* = 0.001), Genchi test (*p* = 0.001), prone shoulder flexion (*p* = 0.003), side lift (*p* < 0.01), roll-up with bent legs (*p* = 0.041), roll-up with straight legs (*p* = 0.004), leg pull (*p* = 0.003), and push-up on single knee (*p* = 0.034). No significant changes were reported in the control group for these outcomes where reported.
Kalkan-Balak et al. (2024) [20]	Body composition: muscle weight, fat weight; Balance: Functional Reach Test; Muscular strength: modified push-ups.	No significant differences were found for muscle weight in the control group (*p* = 0.993) or Pilates group (*p* = 0.420), while WBV showed a significant change (*p* = 0.040). No significant differences were found for fat weight in the control group (*p* = 0.30), Pilates group (*p* = 0.24), or WBV group (*p* = 0.51). Significant improvements were reported for Functional Reach Test and modified push-ups in the control group, Pilates group, and WBV group (*p* < 0.010).
Hushmandi et al. (2023) [13]	Anthropometry: pelvis circumference.	Significant improvement was observed in the Pilates group (*p* < 0.001), while no significant change was found in the control group (*p* = 0.29).
Tolnai et al. (2016) [16]	Balance: Functional Reach Test; Muscular endurance/strength: static plank.	Significant between-group differences were reported for Functional Reach Test (*p* = 0.001) and static plank (*p* = 0.001), in favour of the Pilates group.
Ceviz et al. (2025) [11]	Muscular strength: back strength, leg strength.	A significant between-group difference was reported for back strength (*p* = 0.004), in favour of the Pilates group. No statistically significant between-group difference was found for leg strength (*p* = 0.083).
Vieira et al. (2017) [15]	Balance: one-leg stance; Functional performance: Timed Up and Go test, sit-to-stand test, 6 min walk test.	No significant differences were found for one-leg stance or TUG performance; *p*-values were not reported. Time effects were observed for sit-to-stand (*p* = 0.05) and 6 min walk test (*p* < 0.01).
Lee et al. (2016) [10]	Muscular strength: lumbar strength, trunk lift.	Significant improvements were reported for lumbar strength and trunk lift in both the Pilates and control groups (*p* < 0.001).

(WBV = whole-body vibration group; TUG = Timed Up and Go test; ROM = range of motion).

The risk of bias assessment for randomized studies showed that two studies [5,21] were judged to have an overall low risk of bias across the evaluated domains, while the remaining studies were mostly classified as having some concerns. These concerns were most frequently related to the randomization process (D1), deviations from intended interventions (D2), and selection of the reported result (D5). Several studies [9,11,20,22] presented methodological uncertainties in multiple domains, resulting in an overall judgement of some concerns. The domain most commonly assessed as low risk was bias due to missing outcome data (D3), where most studies demonstrated adequate reporting. One study, Şavkın [12], showed a high risk of bias in the D2 domain. Overall, although some studies demonstrated low risk of bias, a considerable number of included randomized studies presented methodological concerns that should be considered when interpreting the findings. The complete assessment is presented in Table 4.

**Table 4:** Risk of bias for RCT studies.

Study	D1	D2	D3	D4	D5	Overall
Aibar-Almazán et al. (2022) [5]	Low	Low	Low	Low	Low	Low
Ceviz et al. (2025) [11]	Some concerns	Some concerns	Low	Some concerns	Some concerns	Some concerns
Kalkan-Balak et al. (2024) [20]	Some concerns	Some concerns	Low	Low	Some concerns	Some concerns
dos Reis et al. (2024) [21]	Low	Low	Low	Low	Low	Low
Sekendiz et al. (2007) [9]	Some concerns	Some concerns	Some concerns	Some concerns	Some concerns	Some concerns
Vieira et al. (2017) [15]	Low	Some concerns	Some concerns	Low	Low	Some concerns
Ružić (2020) [22]	Some concerns	Some concerns	Low	Some concerns	Some concerns	Some concerns
Şavkın et al. (2017) [12]	Some concerns	High	Low	Some concerns	Some concerns	High

(D1—Bias arising from the randomization process; D2—Bias due to deviations from intended interventions; D3—Bias due to missing outcome data; D4—Bias in measurement of the outcome; D5—Bias in selection of the reported result; Low—low risk of bias; Some concerns—some concerns; High—high risk of bias).

The risk of bias assessment for non-randomized studies indicated that most studies demonstrated a moderate overall risk of bias. Several studies [10,13,14] showed moderate risk across multiple domains, particularly in relation to confounding and participant selection, while generally demonstrating lower risk in domains related to classification of interventions and missing data. One study, Tolnai [16], showed a serious overall risk of bias, with serious concerns identified in several domains, including confounding, participant selection, deviations from intended interventions, missing data, and measurement of outcomes. Overall, although some domains were assessed as having low risk of bias, methodological limitations were present in most non-randomized studies and should be considered when interpreting the findings derived from these studies. The complete assessment is presented in Table 5.

**Table 5:** Risk of Bias for Non-RCT studies.

Study	D1	D2	D3	D4	D5	D6	D7	Overall
Hushmandi et al. (2023) [13]	Moderate	Moderate	Low	Moderate	Low	Moderate	Moderate	Moderate
Kolomiitseva et al. (2022) [14]	Moderate	Moderate	Low	Moderate	Moderate	Moderate	Moderate	Moderate
Tolnai et al. (2016) [16]	Serious	Serious	Low	Serious	Serious	Serious	Moderate	Serious
Lee et al. (2016) [10]	Moderate	Moderate	Low	Moderate	Low	Moderate	Moderate	Moderate

(D1—Bias due to confounding; D2—Bias due to selection of participants; D3—Bias in classification of interventions; D4—Bias due to deviations from intended interventions; D5—Bias due to missing data; D6—Bias in measurement of outcomes; D7—Bias in selection of the reported result; Low—low risk of bias; Moderate—moderate risk of bias; Serious—serious risk of bias).

**Figure 2:**
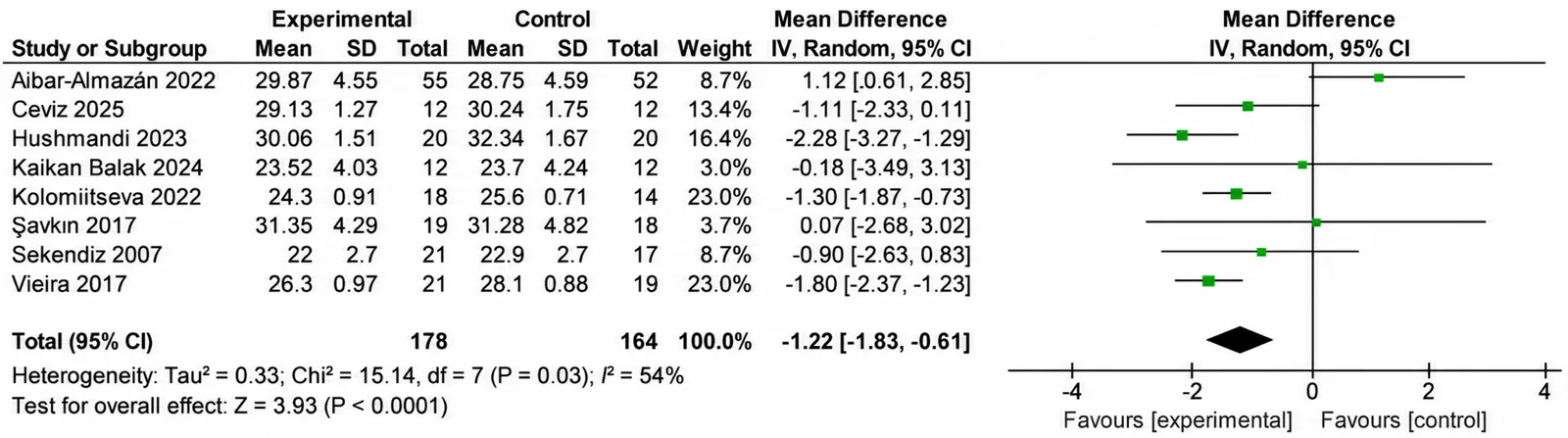
Forest plot of the meta-analysis examining the effect of Pilates interventions on Body Mass Index [5,9,11,12,13,14,15,20].

The meta-analysis of eight studies examined the effects of Pilates training programs on body mass index (BMI), including 178 participants in the experimental groups and 164 participants in the control groups. Using a random-effects model, the pooled analysis showed a statistically significant reduction in BMI in favor of the Pilates intervention (MD = −1.22; 95% CI: −1.83 to −0.61; *p* < 0.001) (see Figure 2). Moderate heterogeneity was observed among the included studies (I^2^ = 54%), indicating some variability in effect sizes across studies; however, the overall result consistently favored the experimental groups.

**Figure 3:**
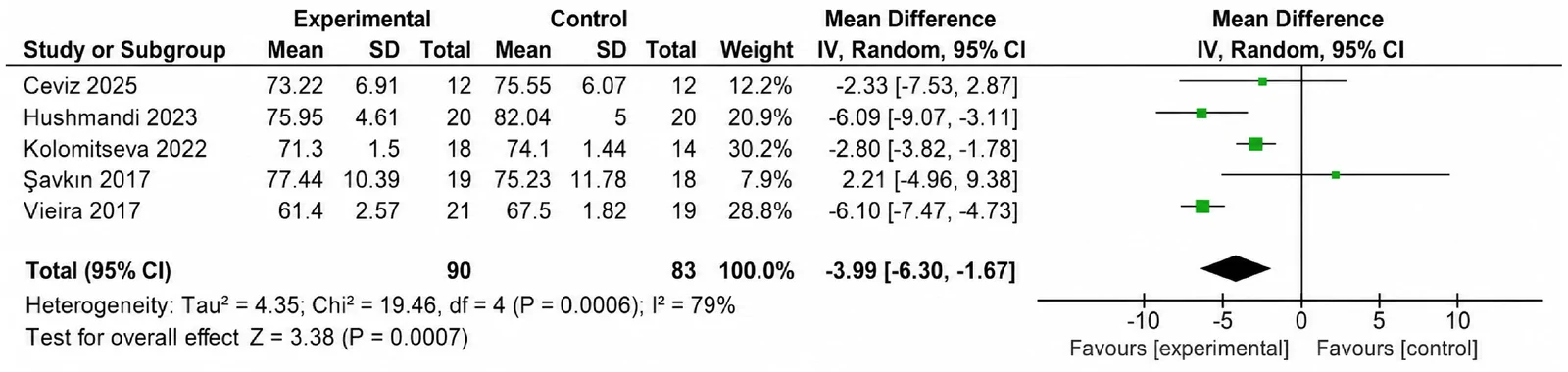
Forest plot of the meta-analysis examining the effect of Pilates interventions on body weight [11,12,13,14,15].

The meta-analysis of five studies examined the effects of Pilates training programs on body weight, including 90 participants in the Pilates groups and 83 participants in the control groups. Using a random-effects model, the pooled analysis showed a statistically significant reduction in body weight in favor of the Pilates intervention (MD = −3.99 kg; 95% CI: −6.30 to −1.67; *p* = 0.0007) (see Figure 3). High heterogeneity was observed among the included studies (I^2^ = 79%), indicating substantial variability in effect sizes; nevertheless, the overall pooled effect favored the experimental groups.

**Figure 4:**
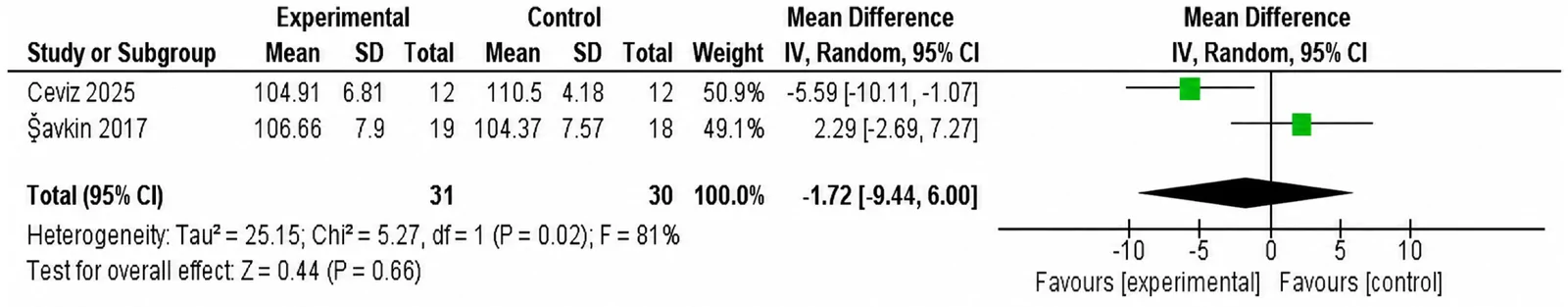
Forest plot of the meta-analysis examining the effect of Pilates interventions on hip circumference [11,12].

The meta-analysis of two studies examined the effects of Pilates training programs on hip circumference, including 31 participants in the Pilates groups and 30 participants in the control groups. Using a random-effects model, the pooled analysis showed no statistically significant effect of Pilates on hip circumference (MD = −1.72 cm; 95% CI: −9.44 to 6.00; *p* = 0.66) (see Figure 4). High heterogeneity was observed among the included studies (I^2^ = 81%), indicating considerable variability in effect sizes and limiting the reliability of the pooled estimate.

**Figure 5:**
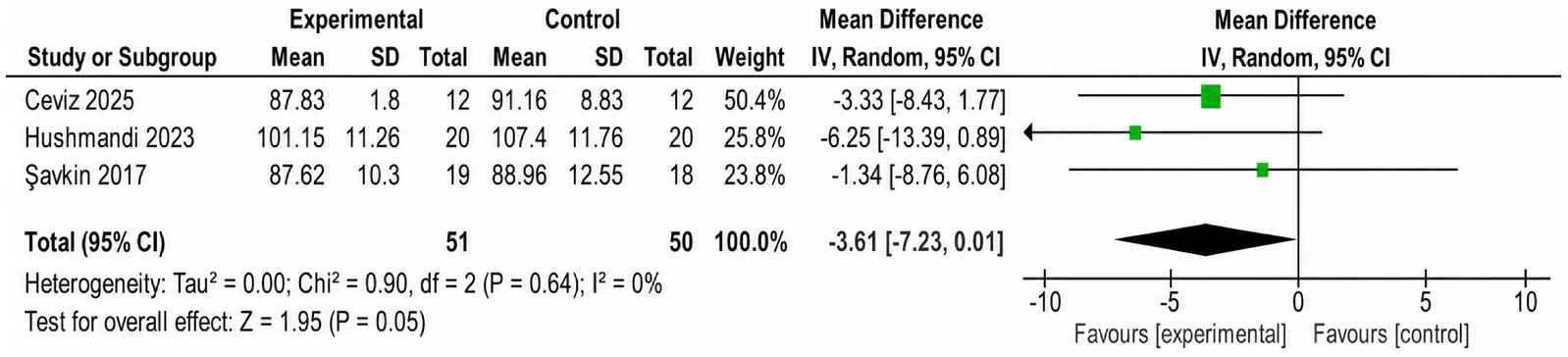
Forest plot of the meta-analysis examining the effect of Pilates interventions on waist circumference [11,12,13].

The meta-analysis of three studies examined the effects of Pilates training programs on waist circumference, including 51 participants in the Pilates groups and 50 participants in the control groups. Using a random-effects model, the pooled analysis did not show a statistically significant effect of Pilates on waist circumference (MD = −3.61 cm; 95% CI: −7.23 to 0.01; *p* = 0.05) (see Figure 5). No heterogeneity was observed among the included studies (I^2^ = 0%), indicating a high level of consistency across the pooled results.

**Figure 6:**
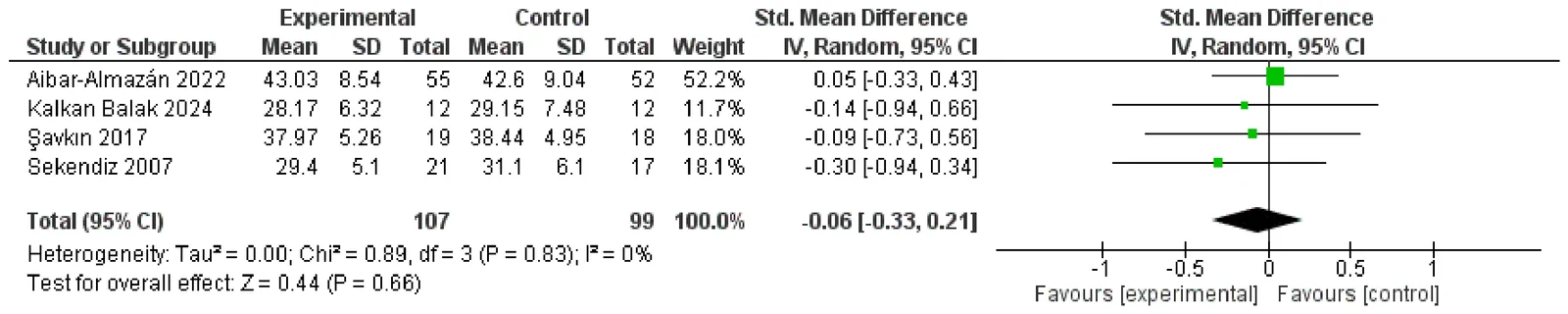
Forest plot of the meta-analysis examining the effect of Pilates interventions on the percent of body fat [5,9,12,20].

The meta-analysis of four studies examined the effects of Pilates training programs on body fat percentage, including 107 participants in the Pilates groups and 99 participants in the control groups. Using a random-effects model, the pooled analysis showed no statistically significant effect of Pilates on body fat percentage (SMD = −0.06; 95% CI: −0.33 to 0.21; *p* = 0.66) (see Figure 6). No heterogeneity was observed among the included studies (I^2^ = 0%), indicating a high level of consistency across the pooled results.

**Figure 7:**
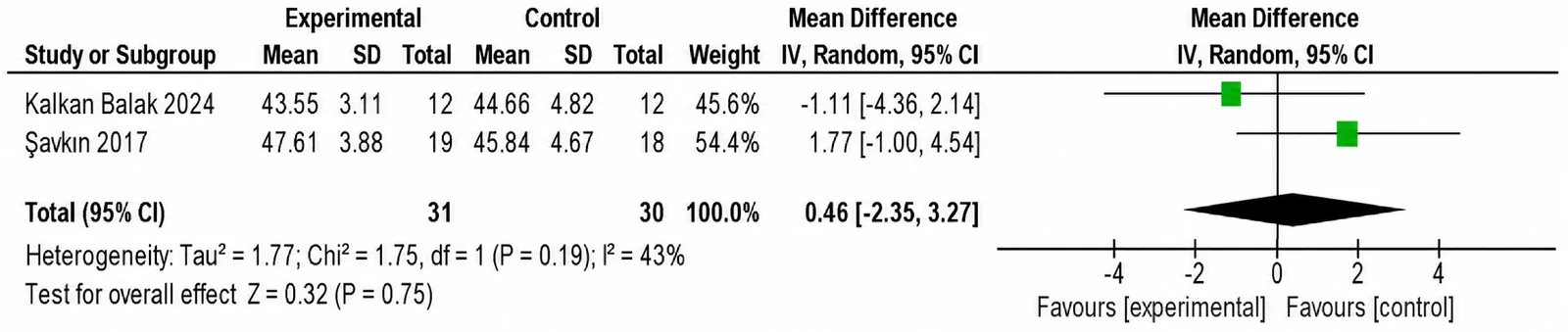
Forest plot of the meta-analysis examining the effect of Pilates interventions on Lean body mass [12,20].

The meta-analysis of two studies examined the effects of Pilates training programs on lean body mass, including 30 participants in the Pilates groups and 30 participants in the control groups. Using a random-effects model, the pooled analysis showed no statistically significant effect of Pilates on lean body mass (MD = 0.46 kg; 95% CI: −2.35 to 3.27; *p* = 0.75) (see Figure 7). Moderate heterogeneity was observed among the included studies (I^2^ = 43%), indicating some variability in the reported effects.

**Figure 8:**
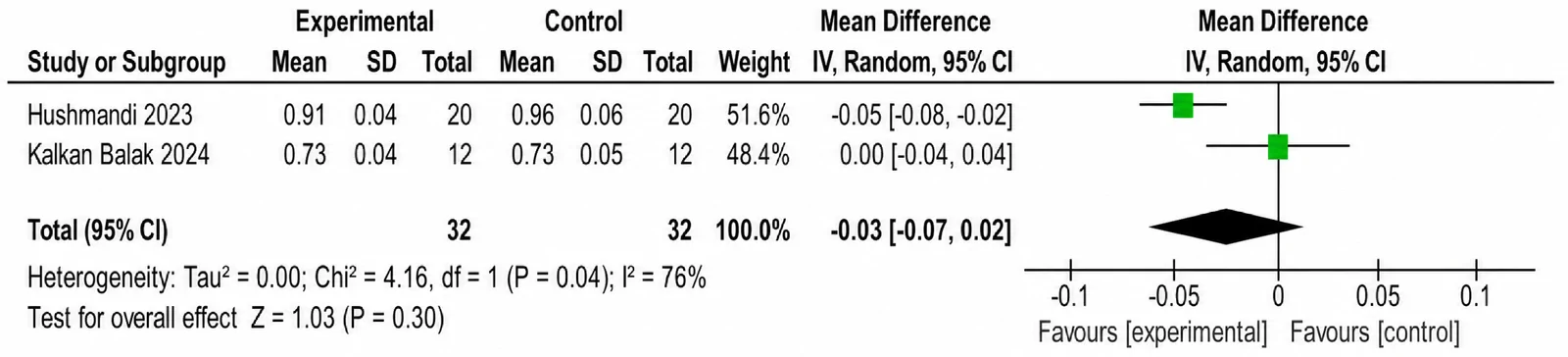
Forest plot of the meta-analysis examining the effect of Pilates interventions on the waist-to-hip ratio [13,20].

The meta-analysis of two studies examined the effects of Pilates training programs on waist-to-hip ratio, including 32 participants in the Pilates groups and 32 participants in the control groups. Using a random-effects model, the pooled analysis showed no statistically significant effect of Pilates on waist-to-hip ratio (MD = −0.03; 95% CI: −0.07 to 0.02; *p* = 0.30) (see Figure 8). High heterogeneity was observed among the included studies (I^2^ = 76%), indicating substantial variability in the reported effects.

**Figure 9:**
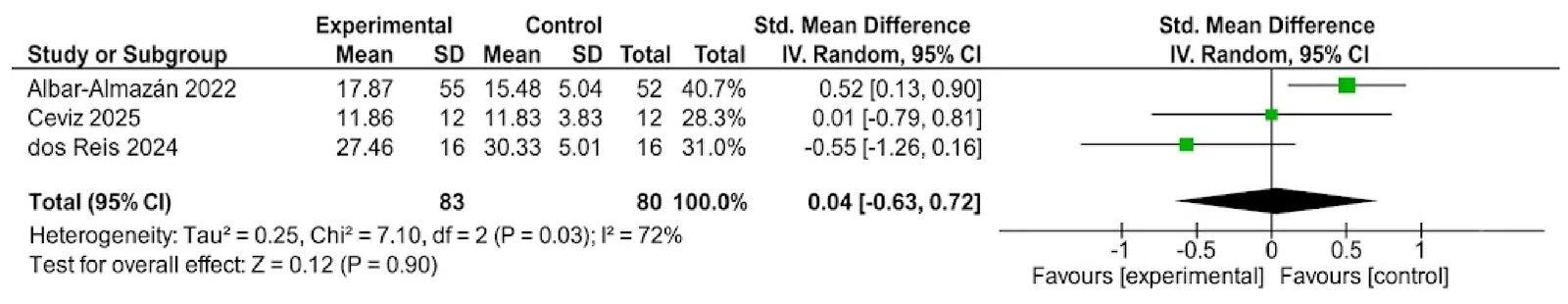
Forest plot of the meta-analysis examining the effect of Pilates interventions on Handgrip strength [5,11,21].

The meta-analysis of three studies examined the effects of Pilates training programs on handgrip strength, including 83 participants in the Pilates groups and 80 participants in the control groups. Using a random-effects model, the pooled analysis showed no statistically significant effect of Pilates on handgrip strength (SMD = 0.04; 95% CI: −0.63 to 0.72; *p* = 0.90) (see Figure 9). High heterogeneity was observed among the included studies (I^2^ = 72%), indicating considerable variability in the magnitude of the reported effects.

**Figure 10:**
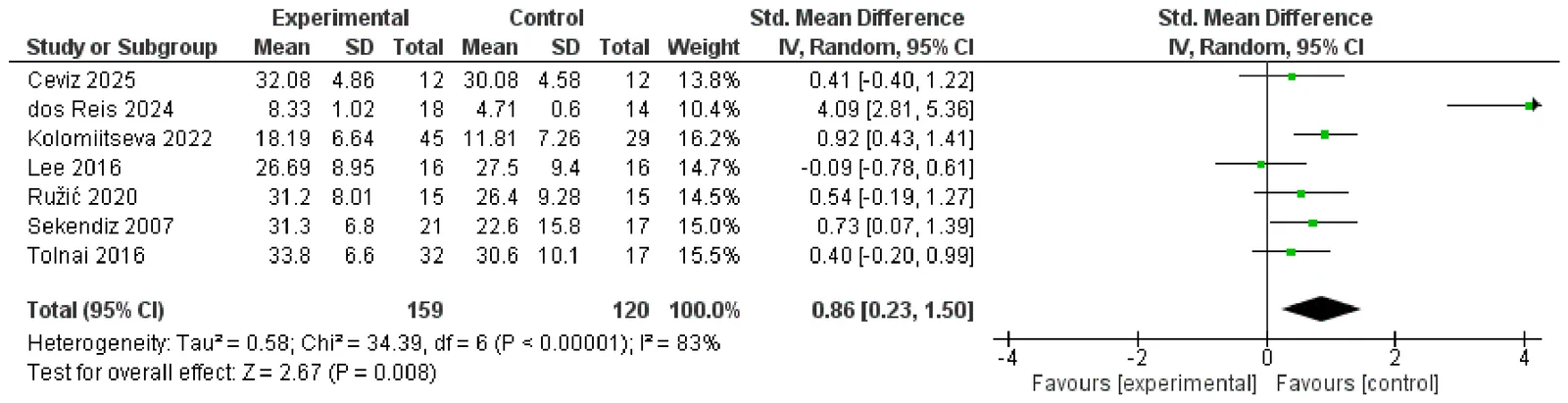
Forest plot of the meta-analysis examining the effect of Pilates interventions on the Sit-and-Reach test. The arrow indicates that the 95% confidence interval extends beyond the displayed range of the forest plot [9,10,11,14,16,21,22].

The meta-analysis of seven studies examined the effects of Pilates training programs on flexibility measured using the sit-and-reach test, including 159 participants in the Pilates groups and 120 participants in the control groups. Using a random-effects model, the pooled analysis showed a statistically significant improvement in flexibility in favor of the Pilates intervention (SMD = 0.69; 95% CI: 0.43 to 0.94; *p* < 0.001) (see Figure 10). High heterogeneity was observed among the included studies (I^2^ = 83%), indicating substantial variability in the reported effects; therefore, the pooled estimate should be interpreted with caution.

**Figure 11:**
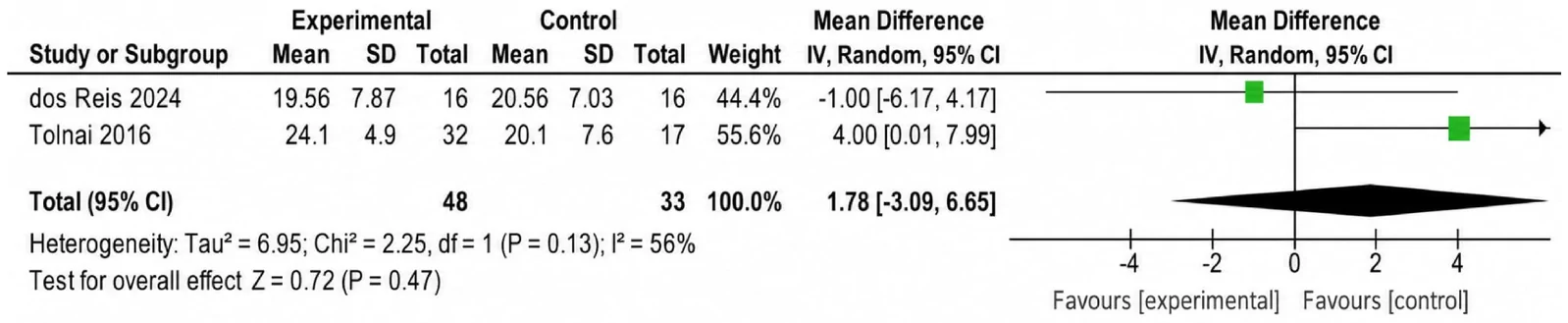
Forest plot of the meta-analysis examining the effect of Pilates interventions on the sit-up test. The arrow indicates that the 95% confidence interval extends beyond the displayed range of the forest plot [16,21].

The meta-analysis of two studies examined the effects of Pilates training programs on functional performance assessed by the sit-up test, including 48 participants in the Pilates groups and 33 participants in the control groups. Using a random-effects model, the pooled analysis showed no statistically significant effect of Pilates on sit-up performance (MD = 1.78 repetitions; 95% CI: −3.09 to 6.65; *p* = 0.47) (see Figure 11). Moderate heterogeneity was observed among the included studies (I^2^ = 56%), indicating variability in the magnitude of the reported effects.

**Figure 12:**
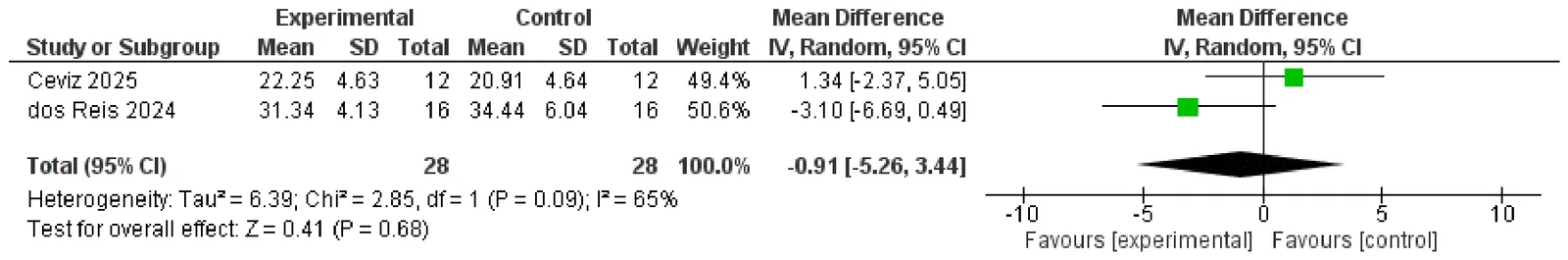
Forest plot of the meta-analysis examining the effect of Pilates interventions on Vertical Jump [11,21].

The meta-analysis of two studies examined the effects of Pilates training programs on vertical jump performance, including 28 participants in the Pilates groups and 28 participants in the control groups. Using a random-effects model, the pooled analysis showed no statistically significant effect of Pilates on vertical jump performance (MD = −0.91 cm; 95% CI: −5.26 to 3.44; *p* = 0.68) (see Figure 12). Substantial heterogeneity was observed among the included studies (I^2^ = 65%), indicating variability in the reported effects and suggesting that the pooled estimate should be interpreted with caution.

### 3.3. Qualitative Analysis of Findings Not Included in the Meta-Analysis

Findings that were not included in the meta-analysis were synthesized narratively according to outcome domain, distinguishing where reported between within-group changes and true between-group (Pilates versus comparator) effects, and are presented in Table 3. For body composition, no significant between-group differences were found for skeletal muscle mass, muscle weight, or fat weight [1,19], a pattern consistent with the non-significant pooled effects for body fat percentage and lean body mass obtained in the meta-analysis. Anthropometric findings were less consistent and, in one case, ambiguous: pelvic circumference improved only in the Pilates group [18], supporting a Pilates-specific effect, whereas abdomen circumference improved significantly in both the Pilates and control groups [12]. Because the change occurred in both arms, it cannot be attributed to the intervention on the basis of the data extracted, and the original study would need to be consulted to determine whether the magnitude of change differed between groups.

For muscular strength and endurance, the evidence is more consistent with a genuine Pilates-specific effect for some outcomes than for others. Between-group differences favoring the Pilates group were reported for the curl-up test and knee flexion/extension strength [9], back strength [11], and static plank and Functional Reach Test performance [16], findings drawn mainly from randomized trials assessed at low or “some concerns” overall risk of bias. By contrast, improvements in 10-RM knee extension [21] and in lumbar strength and trunk lift [10] were reported in both the Pilates and control arms, so these gains are better explained by testing familiarization or the comparator activity itself than by a specific effect of Pilates; the Sorensen test and leg strength, meanwhile, improved in neither group [11,21]. Functional Reach Test and modified push-up performance also improved to a similar degree across the control, Pilates, and whole-body-vibration arms of a third trial [20], again limiting attribution to Pilates specifically. Flexibility outcomes followed a comparable pattern: prone shoulder flexion improved only in the Pilates group [14], whereas the straddle test improved in both the Pilates and control groups [22], and leg lift improved in neither. Reporting these outcomes only as “significant improvement”, without specifying which comparison the significance refers to, therefore overstates the strength of the evidence for a Pilates-specific effect on muscular fitness and flexibility.

Functional performance and balance findings were similarly dependent on which comparison was actually tested. Back leg lift improved only in the Pilates group [22], and side-lift and roll-up performance improved in the Pilates group of a single non-randomized trial for which matching control data were not reported for most outcomes [14]. Timed Up and Go performance, by contrast, did not differ between groups in either study that assessed it [5,15], and improvements in sit-to-stand and 6 min walk-test performance in a third study were described only as within-group “time effects”, without a formal between-group comparison [15]—a considerably weaker form of evidence than a demonstrated group difference; one-leg stance showed no between-group difference in the same study, with *p*-values not reported. Respiratory function evidence rests entirely on the same single non-randomized study, assessed at overall moderate risk of bias [14], in which vital capacity, the Stange test, and the Genchi test improved in the Pilates group while respiratory rate did not change; because this domain was evaluated in only one study, without a demonstrated advantage over a comparator group, it cannot support a generalizable conclusion about the effect of Pilates on respiratory function and should be treated as hypothesis-generating rather than confirmatory. Taken together, the narrative evidence for muscular strength, balance, functional performance, and respiratory outcomes is more limited and less uniform than a simple count of “significant” findings suggests: several apparent improvements were shared by comparator groups, most originate from single small studies with some or serious risk-of-bias concerns (see Table 4 and Table 5), and formal between-group testing was not always performed or reported.

### 3.4. Certainty of Evidence

Due to the mixture of randomised and non-randomised study designs pooled across outcomes, the overall GRADE score ranged from very low to moderate certainty, with the majority of anthropometric and body-composition outcomes rated as very low or low. Of note, evidence was not downgraded regarding indirectness in any of the assessed outcomes, as all included studies involved comparable adult populations undergoing structured Pilates training. Conversely, evidence was most commonly downgraded due to substantial statistical heterogeneity among studies that evaluated body-composition outcomes, as well as due to imprecision arising from small sample sizes across most secondary outcomes. Publication bias could not be formally estimated for any outcome, as fewer than ten studies contributed to each variable. No quality of evidence was upgraded with respect to the values of effect size, regardless of whether MD or SMD was taken into account. More details pertaining to the certainty of evidence assessment of each outcome are given in Table 6.

**Table 6:** Assessment of certainty of evidence.

Outcome	Number of Studies	Risk of Bias #	Inconsistency †	Indirectness ‡	Imprecision §	Publication Bias ¶	Participants (*n*)	Effect Size Measure (95% CI)	Overall GRADE Score
Body mass index (BMI)	8	○	○ (I^2^ = 54%)	⊕	○	NA	342	MD = −1.22 kg/m^2^ (−1.83 to −0.61)	⊕○○○ VERY LOW
Body weight	5	○	○ (I^2^ = 79%)	⊕	○	NA	173	MD = −3.99 kg (−6.30 to −1.67)	⊕○○○ VERY LOW
Handgrip strength	3	⊕	○ (I^2^ = 72%)	⊕	○	NA	163	SMD = 0.23 (−0.08 to 0.54)	⊕⊕○○ LOW
Sit and Reach test	7	⊕	○ (I^2^ = 83%)	⊕	⊕	NA	279	SMD = 0.86 (0.23 to 1.50)	⊕⊕⊕○ MODERATE
Lean mass	2	⊕	○ (I^2^ = 83%)	⊕	○	NA	61	MD = 0.46 (−2.35 to 3.27)	⊕⊕○○ LOW
Vertical jump	2	○	○ (I^2^ = 65%)	⊕	○○	NA	56	MD = −0.96 cm (−3.53 to 1.62)	⊕○○○ VERY LOW
Hip circumference	2	○	○ (I^2^ = 81%)	⊕	○○	NA	61	MD = −1.72 cm (−9.44 to 6.00)	⊕○○○ VERY LOW
Waist-to-hip ratio (WHR)	2	○	○ (I^2^ = 76%)	⊕	○○	NA	64	MD = −0.03 (CI −0.07 to 0.02)	⊕○○○ VERY LOW
Waist circumference	3	○	⊕ (I^2^ = 0%)	⊕	○	NA	101	MD = −3.61 cm (−7.23 to 0.01)	⊕⊕○○ LOW
Body fat percentage	4	○	⊕ (I^2^ = 0%)	⊕	○	NA	206	SMD = −0.06 (−0.33 to 0.21)	⊕⊕○○ LOW
Sit-up test	2	○	○ (I^2^ = 56%)	⊕	○○	NA	81	MD = 1.78 reps (−3.09 to 6.65)	⊕○○○ VERY LOW

Legend: #—Risk of bias was assessed using RoB 2 (randomised studies) and ROBINS-I (non-randomised studies); evidence was not downgraded (⊕) where studies were predominantly at low risk of bias, and downgraded by one level (○) where a mixture of randomised and non-randomised studies was pooled and ROBINS-I identified an overall serious risk of bias (mainly due to confounding and selection of participants), or where blinding of outcome assessors was unclear for performance-based measures. †—Inconsistency was not downgraded (⊕) where I^2^ indicated low/moderate heterogeneity and studies were consistent in the direction of effect, and downgraded by one level (○) for substantial statistical heterogeneity (I^2^ ≥ 50%) not explained by the available subgroup or sensitivity analyses. ‡—No serious indirectness was identified for any outcome, as all included studies involved adult participants undergoing structured Pilates training and directly assessed the outcome of interest using comparable, validated measures. §—Imprecision was not downgraded (⊕) where the confidence interval was narrow and did not cross the line of no effect; downgraded by one level (○) where the pooled estimate rested on a small number of studies/participants and/or a wide confidence interval; downgraded by two levels (○○) based on very few studies and a very small total sample size, with a very wide confidence interval crossing the line of no effect and a non-significant pooled result. ¶—Publication bias could not be formally assessed (funnel plot/Egger’s test not performed) because fewer than 10 studies contributed to any outcome; rated as “NA” (not applicable/not assessable) rather than confirmed absent. ⊕—Evidence not downgraded for that domain; ○—Evidence downgraded by one level for that domain (two consecutive ○○ symbols within a cell indicate a two-level downgrade for that domain). ⊕⊕⊕○—Moderate certainty of evidence; ⊕⊕○○—Low certainty of evidence; ⊕○○○—Very low certainty of evidence. MD, mean difference; SMD, standardised mean difference; CI, confidence interval; NA, not applicable; BMI, body mass index; WHR, waist-to-hip ratio; GRADE, Grading of Recommendations Assessment, Development and Evaluation.

## 4. Discussion

### 4.1. Comparison with Existing Knowledge

The results obtained are partly consistent with the findings of previous systematic reviews. For example, Cruz-Ferreira et al. [6] concluded in their review that Pilates can lead to improvements in flexibility and muscular endurance, but they also highlighted the limited methodological quality of many of the included studies. Similarly, Aladro-Gonzalvo et al. [7] concluded that there is limited evidence to support a significant effect of Pilates training on body composition. In line with these findings, the results of the present review indicate improvements in flexibility as well as in certain body composition parameters, although the magnitude and consistency of these effects varied across studies.

### 4.2. Data Summary

This systematic review and meta-analysis aimed to examine the impact of Pilates exercise programs on health-relevant components of physical fitness in adult women. The results of the meta-analysis suggest that Pilates interventions may positively affect certain body composition and flexibility parameters. Statistically significant improvements were observed in body mass index (BMI), body mass, and flexibility measured by the sit-and-reach test. Although the title refers to adult women, the included studies encompass heterogeneous populations (e.g., young women, college students, postmenopausal women, obese and sedentary women). The implications of this clinical heterogeneity should be more explicitly acknowledged in the Discussion. However, these findings should be interpreted with caution due to the clinical and methodological heterogeneity among the included studies, particularly differences in participant characteristics, Pilates modalities, intervention duration and frequency, and comparator conditions, which may limit the generalizability of the results to all adult women. On the other hand, the results did not show statistically significant changes in body fat percentage, waist circumference, waist—to—hip ratio, handgrip strength, explosive lower limb strength (vertical jump), or lean body mass.

The reduction in body mass index and body mass index may be explained by the energy demands of Pilates training, which involves continuous activation of large muscle groups through controlled movements and trunk stabilization. This interpretation is supported by individual studies included in this review that reported favourable changes in body composition and anthropometric indices following Pilates training in sedentary, middle-aged, and obese women [11,12,13]. Although Pilates is often classified as moderate-intensity exercise, regular performance of these exercises may contribute to increased energy expenditure and positive adaptations in metabolic processes. These effects may be particularly pronounced in populations with lower baseline levels of physical activity, such as sedentary or physically inactive women who were often included in the studies analyzed.

The significant improvement in flexibility confirms the basic principles of the Pilates method, which is based on movement control, stabilization of the core muscles and increased joint mobility. Exercises in Pilates programs often include static and dynamic stretching, as well as controlled movements through a wide range of motion, which can lead to improvements in flexibility and mobility. These results are consistent with previous research indicating that Pilates training can significantly improve flexibility and functional abilities [6,9,22].

In contrast, the results of this meta-analysis did not show significant changes in some fitness parameters, such as handgrip strength, vertical jump, or lean body mass. One possible explanation may be the relatively low level of external loading that characterizes most Pilates programs. Unlike resistance training or strength training, Pilates exercises rely mainly on your own body weight and controlled movements, which may not be sufficient to increase muscle mass or explosive power significantly. This is consistent with previous reviews reporting limited evidence for a significant effect of Pilates on body composition and strength-related outcomes [7].

### 4.3. Strengths and Limitations

The present study has several strengths. First, it was conducted in accordance with PRISMA 2020 guidelines and was prospectively registered in PROSPERO, which enhanced methodological transparency and reduced the risk of selective reporting. Second, both quantitative and narrative syntheses were performed, allowing a comprehensive overview of the available evidence. Third, the review focused specifically on adult women, providing population-specific conclusions regarding the effects of Pilates training on health-related physical fitness. Finally, multiple outcomes related to body composition, muscular fitness, and flexibility were examined, offering a broad perspective on the potential benefits of Pilates interventions.

Several limitations of the present study should be considered when interpreting the findings. First, the number of included studies for certain outcomes was relatively small, and many of the individual studies were conducted with limited sample sizes, which may have reduced the statistical power of the meta-analyses and increased the likelihood of imprecise effect estimates. Second, substantial clinical and methodological heterogeneity was observed across the included studies. Participants differed in age, training status, health status, and baseline physical fitness, while intervention protocols varied in duration, weekly frequency, session length, exercise modality (mat-based or equipment-based Pilates), level of supervision, and training intensity. Such variability may have influenced the pooled results and limits the comparability of findings across studies.

Third, several studies raised concerns regarding risk of bias, particularly in relation to randomization procedures, allocation concealment, blinding, incomplete outcome data, and selective reporting. These issues were more pronounced in non-randomized studies, where the influence of confounding factors and participant selection bias cannot be excluded.

Several pooled analyses exhibit substantial heterogeneity (e.g., body weight, flexibility, and handgrip strength). The Discussion would benefit from a more detailed explanation of the potential sources of heterogeneity, including differences in Pilates modality, intervention duration, participant characteristics, and comparator groups. Fourth, the substantial heterogeneity observed in several pooled analyses, particularly for body weight, flexibility, and handgrip strength, may be explained by differences in the characteristics of the included interventions and participants. Pilates programs varied in terms of modality, exercise selection, training frequency, and intervention duration, while participants differed in age, baseline physical fitness, body composition, and habitual physical activity levels. In addition, differences in comparator conditions, including usual care, no intervention, or alternative exercise programs, may have contributed to variability in the observed effects. These clinical and methodological differences should therefore be considered when interpreting the pooled estimates and may partly explain the variability in treatment effects across studies.

A potential limitation of this review is the insufficient consideration of dietary intake and other factors that may influence the assessed outcomes. Dietary habits and energy intake could potentially affect body composition, body weight, and other health-related outcomes, but these factors were not consistently reported or controlled across the included studies. Therefore, the possible influence of dietary intake should be considered when interpreting the findings of this review.

Therefore, the findings of the present study should be interpreted with caution, and future high-quality randomized controlled trials with larger samples and standardized protocols are warranted.

### 4.4. Future Directions

Future research should focus on conducting high-quality randomized controlled trials with larger sample sizes and standardized training protocols in order to enable more reliable comparisons and more precise estimates of the effects of Pilates interventions. Particular attention should be given to clearer definitions of training intensity, exercise progression, training frequency, and program duration, as these factors may substantially influence the observed adaptations.

It is also important to investigate the long-term effects of Pilates training, including the sustainability of achieved improvements after the intervention period, as well as participant adherence over extended periods of time. Future studies should also examine potential differences in outcomes between various forms of Pilates, such as mat-based Pilates and equipment-based Pilates, as well as between supervised and unsupervised exercise programs.

In addition, it would be valuable to expand research to different female populations, including younger adult women, middle-aged women, postmenopausal women, sedentary individuals, and women with specific health conditions or increased risk of metabolic disorders. Finally, future studies are encouraged to include a broader range of outcomes, not only physical fitness parameters, but also psychological, functional, and quality-of-life measures, in order to provide a more comprehensive understanding of the overall benefits of Pilates training.

### 4.5. Practical Implications

The practical and health significance of the present study lies in providing evidence-based support for the use of Pilates as a safe and accessible form of physical activity for adult women. The findings indicate that Pilates training may contribute to improvements in flexibility and selected body composition parameters, which are important components of overall health and functional fitness. These benefits may help reduce the risk of musculoskeletal discomfort, mobility limitations, and health problems associated with physical inactivity and excess body mass.

From a practical perspective, Pilates programs can be easily adapted to different ages, fitness levels, and training environments, making them suitable for preventive, recreational, and community-based exercise settings. In addition, due to the relatively low impact and low injury risk, Pilates may represent an appropriate exercise option for women who are beginners, previously inactive, or returning to physical activity after a prolonged period of inactivity. Therefore, Pilates can be considered a valuable strategy for promoting long-term health, regular exercise participation, and quality of life in adult women.

## 5. Conclusions

The results of this systematic review and meta-analysis indicate that Pilates training may have a positive effect on certain components of health-related physical fitness in adult women. The most pronounced effects were observed in the reduction in body mass index, body mass, and improvement in flexibility measured by the sit-and-reach test. These findings suggest that Pilates may contribute to improvements in selected body composition parameters and mobility, particularly in women with lower baseline levels of physical activity. From a practical perspective, Pilates may be particularly beneficial for adult women aiming to improve flexibility and selected body composition outcomes; however, due to the variability in intervention duration, frequency, and modality across the included studies, a single optimal Pilates program cannot be established. Future research should focus on identifying the optimal duration, frequency, intensity, and program characteristics for specific populations of adult women. On the other hand, the results did not show statistically significant changes in body fat percentage, waist circumference, waist-to-hip ratio, handgrip strength, explosive lower-limb strength, or lean body mass, indicating that the effects of Pilates are not equally pronounced across all domains of physical fitness.

The practical significance of this study lies in the fact that Pilates may represent a safe, accessible, and adaptable form of physical activity for adult women. Due to its adaptability to different ages, fitness levels, and health statuses, Pilates can be applied in recreational, preventive, and health-oriented exercise programs. However, due to differences in participant characteristics, intervention duration and structure, and the methodological quality of the included studies, the findings should be interpreted with caution. Future research should include larger samples, standardized training protocols, and long-term follow-up in order to more clearly determine the actual contribution of Pilates training to improving women’s health, functional ability, and quality of life.

## Data Availability

The raw data supporting the conclusions of this article will be made available by the authors on request.
